# Interprofessional simulation as an enhancement of psychological fidelity: A focus group study of medical and nursing students

**DOI:** 10.1002/hsr2.1237

**Published:** 2023-05-02

**Authors:** Bryan Burford, Paul Grieg, Mike Kelleher, Clair Merriman, Alan Platt, Elize Richards, Neil Davidson, Gill Vance

**Affiliations:** ^1^ School of Medicine Newcastle University Newcastle upon Tyne UK; ^2^ Nuffield Department of Clinical Neurosciences University of Oxford Oxford UK; ^3^ Department of Nursing, Midwifery & Health Northumbria University Newcastle upon Tyne UK; ^4^ Oxford Institute of Nursing, Midwifery, and Allied Health Research Oxford Brookes University Oxford UK; ^5^ Medical School University of Oxford Oxford UK

**Keywords:** health professions education, interprofessional education, medical education, patient simulation, simulation fidelity

## Abstract

**Background and Aims:**

Interprofessional simulation has the potential to enhance the perceived realism of clinical simulation in the education of different healthcare professionals. This study considers how the inclusion of more than one profession in clinical simulation contributes to this psychological fidelity, defined as the subjective perception of the realism of a simulation, and the cues identified by medical and nursing students.

**Methods:**

Eight focus groups were carried out with 27 medical and 18 nursing students in Newcastle and Oxford, UK. These were carried out immediately after students' participation in simulation sessions consisting of three acute scenarios. Focus group discussions encompassed perceptions of the realism of the simulation and of participants' own and other professional groups. Thematic analysis was carried out on transcripts.

**Results:**

The analysis identified features of psychological fidelity that were influenced by the interprofessional element of the simulation. These included overall impressions of realism, and the perceived roles and expectations of doctors and nurses within the simulation. In particular, the presence of the other professional group afforded a more authentic response. Other features varied with the viewpoint of the student groups, in particular the realism of the patient manikin, which held lower psychological fidelity for the nursing students, because it did not allow them to fulfill their perceived role of delivering holistic, relational care.

**Conclusion:**

Recognizing “psychological fidelity” as a subjective response to simulation allows greater consideration of the limitations of fidelity as a designed or engineered property of a simulation. While interprofessional involvement directly enhances psychological fidelity in some ways, potential differences in the views of students from different professional groups should be considered when implementing interprofessional simulation.

## 1 INTRODUCTION

Clinical simulation as an educational resource has become an integral part of undergraduate curricula for many healthcare professions. While much simulation is uniprofessional, taking place with groups of students within a particular course, literature has considered interprofessional simulation, where members of different clinical professions learn together in acute scenarios.^1^, ^2^, ^3^, ^4^, ^5^ Among the effects of interprofessional simulation are benefits for communication and teamwork, increased awareness of other professions, and improved attitudes towards interprofessional learning.^6^, ^7^, ^8^, ^9^, ^10^, ^11^, ^12^

Delivering interprofessional simulations can have organizational overheads, for example in finding shared time in different curricula for student groups who are often not colocated. However, there is a potential benefit in the additional “realism” brought to a simulation through the involvement of members of another profession. This paper considers the contribution of an interprofessional element to perceptions of simulation fidelity.

### 1.1 Interprofessional involvement and simulation fidelity

The foundation of much simulation design is that the closer a simulation resembles the practice environment‐the higher its fidelity‐the better learning transfer will be. There is mixed evidence as to whether higher fidelity is actually beneficial to learning,^13^, ^14^, ^15^ but it remains a working assumption behind much simulation practice.

Consideration of this topic is confounded by the complexity of the concept and language of fidelity,^16^, ^17^ and authors have suggested it should be abandoned.^18^ Indeed, in developing this paper we spend some time avoiding the term, before reflecting that it is better to embrace commonly used terminology rather than fragment language further.

A typology of fidelity can be identified across the literature, albeit with ambiguities, regarding the ways in which a clinical environment is represented and experienced.^19^, ^20^, ^21^

The terminology used varies,^22^ but in general, *physical* or *structural* fidelity has been used to refer to the extent to which a simulator physically represents the clinical environment, and is perhaps the default criterion on which “high” or “low” fidelity has been judged.^23^ *Conceptual* ^20^ or *functional* ^24^ fidelity on the other hand refers to the extent to which the simulator responds as a real patient, or real equipment, would. *Sociological* fidelity has been coined as a reflection of power and structural issues within the simulated environment.^21^

*Psychological* fidelity describes the extent to which a simulator elicits or affords authentic responses. In some uses, this still refers to designed elements of the simulator‐as‐stimulus,^13^, ^25^, ^26^ which can lead to some circular definition (e.g., “does the simulator contain the critical elements to accurately simulate the specific behaviors required to complete the task?”^13^ ^,p.637^ can be read as a redescription of simulation in toto). We suggest that reserving psychological fidelity to describe the ontologically distinct *response* to a simulator provides more utility. This can be expressed as “the degree to which the trainee perceives the simulation to be a believable surrogate,”^27^ ^,p.i52^ or more simply as the learner's subjective perception of the simulator's realism.

The introduction of other professionals may be expected to increase psychological fidelity, by providing additional cues that the simulation represents an authentic clinical environment (“reality cues”^24^). These cues could be considered environmental or sociological, but beyond the simple introduction of other learners in a scenario they are not in the control of the designer, and so viewing them as a feature of the simulator‐as‐stimulus may limit understanding of their effects.

The response of learners to interprofessional simulation has been considered in the literature, although not always using the language of fidelity. For example, Oxelmark and colleagues^8^ noted a sense of “embodiment” reported by students, in the stress and time pressures experienced in the simulation. However, Rossler and Kimble ^28^ noted that physical therapy students did not feel an interprofessional simulation was “realistic” because their work is not usually in the context of an interprofessional team—here the interprofessional element was a “fiction cue”. Naismith and colleagues^25^ indicate a sense of realism arising from what they view as the sociological fidelity of another profession's involvement.

### 1.2 The current study

In this paper, we present analysis of qualitative data which explored medical and nursing students' perceptions of interprofessional simulation. The analysis sought to answer two research questions around psychological fidelity (defined as the perceived realism, or authenticity, of the simulation):

How does the inclusion of an interprofessional element in clinical simulation affect the psychological fidelity of the simulation?

What features of interprofessional simulation described by medical and nursing students may influence psychological fidelity?

## 2 METHOD

As the phenomenon of interest was learner perceptions, a qualitative study with an interpretivist perspective was undertaken. Eight focus groups were conducted with medical students and student nurses in two areas of the United Kingdom. Data collection took place in two phases—the first consisted of six groups conducted in Newcastle, UK, and the second of two groups—one in Newcastle and one in Oxford, UK. These latter groups were used to examine and validate preliminary analysis of the first focus groups. The inclusion of a second site was intended to indicate whether perceptions of fidelity differed with the context of the simulation.

Participants were also asked to complete questionnaires before and after the sessions. These constituted a distinct study, concerned with separate questions of how interprofessional simulation may affect measures of professional identity and readiness for interprofessional learning. This has been published elsewhere.^10^

### 2.1 Participants and recruitment

Participants were medical students from Newcastle and Oxford universities and nursing students from Northumbria and Oxford Brookes Universities. Newcastle medical students were in a practice‐based block 2–3 months before finals, while Oxford medical students were in a similar block *following* finals. Most nursing students were in their 2nd or 3rd year, although some first years took part.

Students were informed of the research project in advance by teaching faculty, who stressed that participation in the research was voluntary, and that attending the teaching (which was voluntary for nursing students in Newcastle, and for medical students in Oxford) did not mean a commitment to taking part in the research. Following simulation sessions held in the morning, students were invited to take part in a focus group immediately following the session.

### 2.2 Setting and educational context

The conduct and intent of simulation sessions was similar in Newcastle and Oxford. Both locations used high‐fidelity patient simulators in purpose‐built simulation centers, with authentic equipment to represent an acute bay. The clinician authors (AP, MK, GV, ND, PG, ER, CM) had all been involved in the design and/or delivery of the sessions.

Each session comprised three acute scenarios requiring interprofessional collaboration (such as sepsis, anaphylactic reaction, pulmonary embolism). The clinical details varied but the interprofessional aspects were consistent. In each scenario, the initial assessment was conducted by nursing students, who then called medical students playing the role of junior doctors. The medical students conducted their own assessments and began management, working with the nurses to monitor observations, organize investigations, administer drugs, and so forth. The patient would then deteriorate following a planned protocol. In Newcastle, scenarios were terminated when the students called a senior (played by a member of clinical faculty in the simulation control room) for help while in Oxford the scenarios could continue beyond this point into resuscitation, or even manikin “death”. Each scenario took around 20–40 min to unfold, followed by a 30–40 min debrief facilitated by a member of teaching faculty.

Interprofessional working was not an a priori defined learning outcome of the sessions, nor of the debrief. The focus was on clinical and practical learning around the management of the acute scenarios featured. We see this as a strength of our study with regard to our interest in interprofessional learning, as students were not primed to consider the impact of the other professional group, but rather reflected on the interprofessional aspect as part of their clinical learning.

In Newcastle, medical students entered the scenario in pairs, with one designated “lead” (prescribed in advance by teaching faculty, and not manipulated as part of the research). In Oxford, the lead role was not allocated in advance, but students could agree to this among themselves or allow a lead to emerge during the scenario. Nurse roles developed organically in all settings. The remainder of the groups observed the scenario remotely through a video link, but in Oxford observers could also be called on to enter the scenario and assist.

A member of the simulation faculty was also present in the simulation room. While performed by clinicians, these were professionally neutral roles, providing details of observations that were not available through the patient simulator (e.g., capillary refill time). A member of faculty also played the senior doctor called by students – this was not always played by a doctor.

In some Newcastle sessions, a clinical educator was also present in the observation room, providing commentary and facilitating discussion. A nonclinical researcher (author BB) was also present in some of these, but did not contribute to discussions.

### 2.3 Data collection

Students who wished to stay behind following the simulation session were briefed on the purpose of the focus group, and were asked to provide consent for the discussion to be recorded.

In Newcastle, three groups were conducted with nursing students and four with medical students. In Oxford, a single group consisted of both medical and nursing students (the final group in Newcastle had been intended to be a mixed group, but this proved not to be possible). Groups had between four and eight participants. Group discussions took 45–55 min and were audio‐recorded. Groups in Newcastle were facilitated by BB (nonclinical) and ND (medical), and the group in Oxford by GV (medical).

A topic guide was used to ensure all discussions covered the main areas of interest, although group dynamics varied. This was derived from the research questions and discussed and reviewed by all authors. Questions addressed participants' experiences of interprofessional learning and working, their perceptions of their own and the other professional group, of how real the simulation felt, and whether they “felt like” a doctor or nurse during the simulation, and why. Although a standardized guide (available in the appendix) was used to ensure topics were covered, the specifics of each discussion and prompts used were responsive to individual groups.

Focus groups were audio‐recorded and transcribed by a professional transcription service.

### 2.4 Analysis

An inductive thematic analysis, corresponding to “codebook” thematic analysis,^29^ was led by BB and GV, who familiarized themselves with all transcripts and independently coded two transcripts before agreeing a set of codes to be applied to all the transcripts from the first six focus groups in Newcastle. This coding was undertaken in Microsoft Word by BB, GV, and ND. These codes were then considered against the final two focus groups from Newcastle and Oxford to see if they could be readily applied to a different cohort and a different context. Codes were applicable to these transcripts, indicating coding was appropriate and different topics had not been raised.

Coded text was then sorted in Microsoft Excel, before the sorted codes were copied into new Word documents and further reviewed to identify themes which formed the basis of analytical narratives. All authors contributed to this stage of analysis, and subsequent iterative revision in the development of this manuscript.

While the analysis was inductive, the narrative as presented here is structured to reflect the focus of the research questions, relating to fidelity. We did not directly consider interprofessional differences during the analysis, but in developing the final narratives we considered whether quotes from different groups provided any evidence that professional group membership shaped perceptions.

### 2.5 Reflexivity

The design of the study and interpretation of results was informed by the different professional backgrounds of the authors: nursing (AP, MK, CM), medicine (GV, ND, PG, ER), and nonclinical (BB). The broad range of insight from these different experiential positions allowed assumptions around the roles of doctors and nurses, and the realism of the simulated environment, to be recognized and challenged. Different degrees of involvement in the design and delivery of sessions also provided useful different perspectives on the role of simulation.

### 2.6 Ethical review

Ethical approval was provided by Newcastle University Faculty of Medical Sciences Research Ethics Committee (ref 00856/2015), and this was accepted by the research ethics committees at Northumbria, Oxford, and Oxford Brookes universities.

The COREQ checklist^30^ has been followed in reporting this study, where appropriate.

## 3 RESULTS

In total 26 medical students and 18 nursing students took part in eight focus groups, with between four and eight participants per group. All nurse participants were female, while all medical student groups contained a gender mix (with 12 male and 15 female students across all groups).

The results presented here reflect themes which mapped to the research questions of this paper: indicating features of the interprofessional simulation which either affected participants' perceptions of the realism of the simulation, or where perception was linked to the different perspectives of professional groups.

Within the results, we use the words “realism” and “authenticity” to refer to the participants' views of the simulation, as this reflects their terminology and the focus on their perceptions, rather than “fidelity.”

### 3.1 Interprofessional involvement affected overall perceptions of realism

A simulation is by definition unreal, and the expectation of “something happening” constitutes a fundamental limit on psychological fidelity. As one nursing student noted: “it would be a terrible simulation if you went in and the patient was stable and you didn't have anything to do” (focus group 1, nursing student).

Another fundamental fiction cue, that of being observed during the simulation, was also accentuated for some by interprofessional involvement, and the stress of being observed by strangers. However, one participant felt this anxiety actually *increased* realism by simulating the pressure of risk to the patient.

*I think the anxiety of having people you don't know watching you kind of takes the place of the anxiety that this is a real person that might die if you don't sort it out […] you do feel they are probably quite similar [anxieties] but for different reasons. (Focus group 6, medical student)*

For many participants, the interprofessional element clearly enhanced reality:

*Having other students, you know, it's that full skill mix, and I think it's one of the most realistic sims we've had in three years (Focus group 4, nursing student)*.

One medical student noted how interprofessional involvement provided an ambience—a “buzz”—that contributed to a scenario's authenticity.

*I definitely think the nurses add a whole new dimension because they are talking to the patient, they're doing the obs and it adds a whole buzz to the scenario that would happen. There's lots of things happening at once so it just added an extra real factor. (Focus group 6, medical student)*

The simulation session was often the first time participants had any real contact with the other student group, and their presence may therefore have signified a clinical, rather than an educational, environment.

### 3.2 Interprofessional involvement afforded authentic roles

The most direct contribution of the interprofessional element to psychological fidelity was regarding the roles undertaken within the simulation. The presence of others made their roles feel more real, based on perceived congruence between participants' roles in the simulation and their anticipated roles in practice. This was based partly on observation, and partly on stereotypes derived from colleagues and peers within their own groups—doctors diagnose and lead, nurses address the holistic needs of the patient.

The power relationship between doctors and nurses was apparent in some participants' accounts, with an interprofessional hierarchy being accepted and reinforced with the doctor as de facto leader with ultimate responsibility for patient care (“the buck stopping with you,” focus group 3, medical student). While nurses' skills and autonomy were recognized, “you could definitely see the nursing staff were looking for direction from the doctor” (focus group 3, medical student). Some nursing students confirmed the latter view, in that they would wait for “verification” (focus group 8, nursing student) of their decisions before moving on.

However, while stereotypical roles were not necessarily challenged, observing the other group increased awareness of that group's capabilities and expertise. This was expressed almost with surprise, and this reinforcement of professional differences can be read as adding to the realism of the simulation, compared with uniprofessional experiences.

*[You think] how do they even know all this? (Focus group 4, nursing student)*

*As a doctor sometimes you forget the most basic of things like sitting a patient up if they're breathless and it's tapping into that wealth of experience and expertise. (Focus group 3, medical student)*

There was a mutual reinforcement of professional role, whereby interacting with a “real” doctor or nurse led participants to behave more like a “real” nurse or doctor, creating a virtuous circle of increasing authenticity. Perceiving others' behavior as authentic seemed to provide an affordance or increase a drive to behave authentically in response.

*I think when you started off, especially with the nurses being there, that makes you feel like you're in the role […] My scenario was anaphylaxis so the nurse wanted me to know the dose of the actual adrenaline and then someone told her to actually draw it all up and inject it and I think she was a bit surprised, she was like ‘Whoa, you want me to inject it?' (Focus group 6, medical student)*

This reinforcement through authentic interactions contrasted with experiences of uniprofessional simulation.

*It was more how it would be in practice. It's not just going to be student nurses there and nobody else whereas like you know you're phoning the doctor and you've got them there to assist in that sense it definitely was more realistic and you could take more from it. (Focus group 4, nursing student)*

The expectations of others could elicit authentic behavior even when students had no prior experience of a specific scenario. Some medical students indicated that the simulation felt more real than their experiences in practice, because they could take on a more authentic medical role.

*In some ways I feel it's more real than going on the ward and speaking to a patient. I often feel like I'm pretending [on the ward] […] because you're not making any decisions for that patient, whereas I felt it can be more of a real situation with the sim. (Focus group 3, medical student)*

This expectation or affordance from the other group may also have enhanced a reflexive, or deliberate realism, whereby students indicated the role *felt* more real because they *treated* it as real—effectively psychological fidelity being enhanced by “pretending.” While perhaps not unique to interprofessional simulation, it can be inferred that the scaffolding provided by authentic roles made this less of a stretch.

*I think it depends how much you throw yourself into the whole experience as well. Yeah you're aware that it's not a real person, but if you kind of have in your head this is a real person and treat it and see it [as real] and try and throw yourself into the whole learning experience of that, I think you do get more out of it. (Focus group 3, medical student)*

The dominant feeling was that the roles being performed by medical and nursing students felt realistic, and that working with the other group was a key element of reinforcing that realism. It was notable was that participants often referred to their counterparts as “nurse” and “doctor”, rather than as students. This suggests that they perceived others to be occupying qualified professional roles within the context of the simulation, rather than peripheral student roles.

### 3.3 Differing views of the simulation environment

While medical and nursing students' perceptions of their roles appeared to be similarly reinforced by the simulation context, their perceptions of the environment indicated attention to different cues because of their different clinical experience. Nursing students tended to be familiar with wards and equipment from longer embedded placements during their training, and hence found the simulation setting was less real due to specific differences from a familiar workplace.

*You know where everything is before you start your shift and before you take on that responsibility. (focus group 1, nursing student)*

Conversely, medical students who had less overall clinical experience but more experience moving between clinical settings felt the unfamiliar setting was generally in keeping with what they expected in practice.

*In a few weeks’ time if I get called to a crash call to a ward or something [I will] have to kind of search around a bit more, so it might almost have been useful for them to give us less information if you like, you have to find out for yourselves where to get all your equipment. (Focus group 7, medical student)*

The physical environment is constant, but its perceived realism appears to be variable with the viewpoint of the student group involved.

In contrast, both groups felt that the passage of time was unreal. In the simulation, patients deteriorated more quickly than participants felt they would in real life (although not always based on actual experience), and investigations and results were completed in far less time. The speed of response to phone calls, including the arrival of the medical students, and the response from the senior doctor, was also often felt to challenge this temporal authenticity.

### 3.4 The patient relationship

In all scenarios, the patient was physically represented by a patient simulator manikin and voiced by a member of the faculty. Both medical and nursing students indicated that the patient felt ‘real enough’, with several describing an emotional response to the scenario as if to a real patient.

*I think you still panic as if it's a real person. Yeah, ‘Don't you die on me!’ (Focus group 1, nursing student)*

However, the response to some cues appeared to differ between medical and nursing students. While physical cues were identified by both groups as limited, both for their clinical information, from pulse to pallor, the absence of social cues was particularly apparent to nursing students for whom the relationship with and knowledge of the patient was identified as central to their role.

*Instantly when you look at a real person you get an instant relationship, based on not a lot but you get something straight away. But when you look at a piece of rubber or whatever he's made from, you just can't get that. (Focus group 1, nursing student)*

Nursing students felt the absence of these cues inhibited the holistic care that they saw as the essence of their professional role. Medical students noted the absence of clinical cues, but not these social cues.

## 4 DISCUSSION

This study considered psychological fidelity—defined as the perceived realism of a simulation^27^—in the context of interprofessional simulation. It identified examples of interprofessionalism directly increasing psychological fidelity, and examples of ways in which psychological fidelity varied with professional group.

### 4.1 Interprofessional involvement as an enhancement of psychological fidelity

Cues which increased psychological fidelity were apparent in a number of ways. The mere presence of the other group added to an authentic ambience, while others behaving authentically mutually reinforced the perception of learners' own roles being realistic, especially when compared to uniprofessional simulation.

It is notable that expectations of others' roles, and the consequent judgment of realism, initially drew on stereotypes, including auto‐stereotypes of their own profession,^31^ because of a lack of direct exposure in practice. Exposure in the simulation did not necessarily challenge stereotypes (e.g., the “doctors” were expected to act as “leader”) but did perhaps add nuance to the understanding of the other group.

The defaulting to a structural hierarchy of power between doctor and nurse,^32^ and the perception of this as realistic, can be seen as the reinforcement of the sociological fidelity of the interprofessional context.^18^, ^25^ However it perhaps problematises it as something which cannot be designed or engineered because the perceptions of those relationships are not necessarily in themselves authentic.

### 4.2 Psychological fidelity as a variable response

Other aspects of the simulation, reflecting aspects of physical fidelity (in terms of the perceptions of the simulation center environment), and patient‐focused elements of sociological fidelity (the cues provided by the patient manikin), were responded to differently by medical and nursing students, emphasizing that psychological fidelity is a response shaped by individuals' experiential context. A fiction cue^24^ for one group may be a reality cue for another, or simply not attended to at all.

Nursing students in particular seemed to feel some aspects of patient authenticity were lacking. The representation of the whole patient was important, not just regarding the immediate interaction, but in the absence of an ongoing and unfolding relationship: the patient simulator is an *object* in ways a patient fundamentally is not. The presence of medical students may make nursing students feel they can behave more like nurses, but if the fundamental fiction of the patient representation undermines this reality, its value may be limited.

### 4.3 Relevance for simulation design and future research

These findings clarify the concept of “psychological fidelity” as a subjective phenomenon, distinct from features of the simulation as designed. This has implications for the future development of interprofessional simulation. Viewing psychological fidelity as a response allows us to consider how different cues, whether sequential or concurrent, may increase or decrease the simulated reality of a simulation—the overall fidelity of a simulation is not a constant. Future work could perhaps examine how psychological fidelity varies as a simulation scenario unfolds.

Interprofessional simulation needs to recognize that participants from different professions may not be experiencing the same “reality”. Simulation design needs to consider how psychological fidelity may vary between learner groups, and how cues may be differently salient for different groups.

We have also found that simulation participants themselves can be agents of psychological fidelity—both deliberately through “pretending” to increase the reality of a scenario, and less consciously through responding to the perceived authenticity of others' roles. This affordance of authentic “performance” is perhaps the core strength and relevance of interprofessional simulation, allowing learners to more fully inhabit their own professional role.

### 4.4 Strengths and limitations

As with all studies, there are limitations to ours. Most of our data was collected from one site and at one point in time. However, we found that initial analysis of that data was robust when considered against data collected a year later and in a different setting; this suggests we had achieved data saturation, and adds credibility to our interpretation. The settings varied in some details of the specific clinical scenarios and how sessions were run, but we take this as a positive in that our analysis has also not been bound by specifics of place, curriculum or individual clinical scenarios (which in fact were rarely referred to by participants). This means transferability of findings to other instances of interprofessional simulation is less limited than if our study had been tightly controlled.

The learning outcomes of the simulation sessions did not explicitly include interprofessional learning. We see this as a strength, because participants were not primed to think about the other group as anything other than clinical colleagues. This increases the credibility of their responses relating to those colleagues.

We approached psychological fidelity as something subjectively perceived and experienced, and so best understood through an interpretivist paradigm. This limits the extent to which our identification of differences between the perceptions of nursing and medical students can be assumed to reflect any systematic differences in the student populations. However, we suggest the differences we have identified are based on intrinsic features of nursing and medical practice, albeit as perceived by the participants.

This study did not consider the efficacy of simulation for teaching and learning, and so does not inform judgements of whether high‐fidelity simulation does or does not benefit learning. However, by elaborating what fidelity is, and the limitations on viewing it as a constant property of a given simulation, it may allow more nuanced judgements on the value of different types of simulator to be made.

## 5 CONCLUSION

Interprofessional involvement directly increases the perceived realism of simulation. While students may lack wider experience to know whether the simulation reflects reality, the involvement of another professional group makes it “feel” more real, and encourages them to occupy their professional role more fully.

As different professional groups may perceive elements of simulation authenticity differently, the design of interprofessional simulation should consider how interprofessional viewpoints may differ.

Further work may consider in more detail how different elements of a simulation are perceived by different professional groups, including those beyond medicine and nursing.

## AUTHOR CONTRIBUTIONS

**Bryan Burford**: Conceptualization; data curation; formal analysis; funding acquisition; investigation; methodology; project administration; writing—original draft; writing—review and editing. **Paul Grieg**: Investigation; methodology; project administration; writing—review and editing. **Mike Kelleher**: Investigation; methodology; project administration; writing—review and editing. **Clair Merriman**: Investigation; methodology; project administration; writing—review and editing. **Alan Platt**: Funding acquisition; methodology; project administration; writing—review and editing. **Elize Richards**: Investigation; methodology; project administration; writing—review and editing. **Neil Davidson**: Formal analysis; investigation; methodology; writing—review and editing. **Gill Vance**: Conceptualization; formal analysis; funding acquisition; investigation; methodology; project administration; writing—original draft; writing—review and editing. All authors have read and approved the final version of the manuscript. Bryan Burford had full access to all the data in this study and takes complete responsibility for the integrity of the data and the accuracy of the data analysis.

## CONFLICT OF INTEREST STATEMENT

The authors declare no conflict of interest.

## ETHICS STATEMENT

Ethical approval was provided by Newcastle University Faculty of Medical Sciences Research Ethics Committee (ref 00856/2015).

## TRANSPARENCY STATEMENT

The lead author Bryan Burford affirms that this manuscript is an honest, accurate, and transparent account of the study being reported; that no important aspects of the study have been omitted; and that any discrepancies from the study as planned (and, if relevant, registered) have been explained.

## Data Availability

Data available on request due to privacy/ethical restrictions.

## FOCUS GROUP TOPIC GUIDE

### 1

**Welcome**

[Review information sheet and consent form]

[Start recording]

[Topics should be covered, but question and prompt wording is a guide – discussions may not follow these points in order]

The first thing we're interested in is how real the simulation felt. Did it feel real to you?

Why?

In what ways?

What felt less/more real?

How much did you feel like a [nurse/doctor] in the simulation?

Did you feel like you were doing the job?

Why did you feel like that?

What do you think defines a [doctor/nurse]?

What are the key things that define the profession?

What is the role of a [doctor/nurse] in the clinical workplace?

How does the simulation compare to your experience of practice on wards?

In what way?

Tell us about your clinical experience?

Did having another group there [of medical/nursing students] affect you?

In what way?

Was it good/bad/neutral their being there?

How much experience have you had of working with them in practice?

Was there any difference between scenarios?

Is there anything else about the simulation that we haven't asked about?
